# The impact of a bundle for the prevention of central venous catheter-associated bloodstream infections in an pediatric surgery intensive care unit

**DOI:** 10.3205/dgkh000584

**Published:** 2025-09-23

**Authors:** İlker Devrim, Deniz Ergun, Ayşe Demet Payza, Hincal Ozbakir, Asya Eylem Boztaş Demir, Pelin Kaçar, Mehmet Can, Özkan Okur, Sena Çam, Canan Dinç, Yeliz Oruc, Nuri Bayram, Arzu Şencan

**Affiliations:** 1 Department of Pediatric Infectious Diseases, Dr. Behçet Uz Children’s Diseases and Surgery Training and Research Hospital, İzmir, Turkey; 2 Department of Pediatric Surgery, Dr. Behçet Uz Childre’s Diseases and Surgery Training and Research Hospital, İzmir, Turkey; 3 Infection Control Committee, Dr. Behçet Uz Children’s Diseases and Surgery Training and Research Hospital, İzmir, Turkey

**Keywords:** Central line associated bloodstream infections, CLABSI, bundle strategy, prefilled flushing syringes, needle-free connectors

## Abstract

**Purpose:**

Central line associated bloodstream infections (CLABSI) cause significant morbidity and mortality. Central line (CL) bundles for the prevention of CLABSI are very effective, but there is a paucity of research concerning this in Pediatric Surgery Intensive Care Units (PSICU). The aim of this study was to evaluate the impact of CLB including PFS and mechanical valve type NFC in a pediatric surgery intensive care unit.

**Materials and methods:**

In a retrospective cross-sectional study, the CLABSI rate was compared before and after the introduction of a CL bundle including mechanical valve-type needle-free connectors and prefilled flushing syringes.

**Results:**

Throughout the research period, a total of 1,092 patients were hospitalized at the PSICU and a total of 142 patients (13.0%) with central venous catheter (CVC) was included. During the pre-bundle period, 6 CLABSIs were diagnosed in 509 CVC days, with an overall rate of 11.79 CLABSIs per 1,000 CVC days. After the implementation of the bundle, 1 CLABSI was diagnosed in 621 CVC days with an overall rate of 1.61 CLABSIs per 1,000 CVC days. The CLABSI rate was thus significantly lower rate in the bundle period (p=0.03).

**Conclusion:**

Because the CVC bundle including mechanical valve-type needle-free connectors and prefilled flushing syringes significantly reduced the CLABSI rates in the PSICU, it should be implemented in pediatric surgical ICUs as well as ICUs for other patient groups

## Zusammenfassung

**Hintergrund:**

Zentralvenenkatheter-assoziierte Blutstrominfektionen (CLABSI) verursachen erhebliche Morbidität und Mortalität. Das Zentralvenenkatheter (ZVK)-Bündel zur Vorbeugung von CLABSI ist sehr wirksam, aber die Studienlage in der pädiatrischen Intensivmedizin (PSICU) ist begrenzt.

**Methode:**

In einer restrospektiven Studie wurden die CLABSI-Rate vor und nach der Einführung eines ZVK-Bündels mit nadelfreien Konnektoren mit mechanischem Ventil und vorgefüllten Spülspritzen verglichen.

**Ergebnisse:**

Während des Untersuchungszeitraums wurden 1.092 Patienten auf der pädiatrisch chirurgischen Intensivtherapiestation behandelt und 142 Patienten (13,0%) mit ZVK einbezogen. In der Zeit vor der Einführung des Bündels wurden 6 CLABSI in 509 CL-Tagen diagnostiziert, was einer Gesamtrate von 11,79 CLABSI pro 1.000 Zentralvenenkathetertage entspricht. Nach Einführung des CLABSI-Bündels wurde 1 CLABSI in 621 ZVK-Tagen diagnostiziert, d.h. durch die Einführung des Bündels wurde die CLABSI-Ratesignifikant reduziert (p=0,03).

**Schlussfolgerung:**

Da durch das CLABSI-Bündel mit nadelfreien Konnektoren mit mechanischem Ventil und vorgefüllte Spülspritzen die CLABSI-Raten auf PSICU erheblich reduziert wurden, sollte in PSICU und anderen chirurgischen Intensivtherapiestationen die Bündelstrategie zur Prävention von CLABSI eingeführt werden.

## Introduction

Central line-associated bloodstream infections (CLABSIs) were the most frequently reported healthcare-associated illness in the United States between 2011 and 2014 [1]. The Infusion Nurse Society (INS) and the US Centers for Disease Control and Prevention (CDC) recommended guidelines for the prevention of CLABSI [2], [3]. Earlier studies from our center reported that the implementation of central line bundle (CLB), including Luer access split-septum connector-type needle-free connectors (NFC) to replace the 3-way stopcock (3 WSC) in addition to using prefilled flushing syringes (PFS), was associated with a significant decrease in CLABSI rates in pediatric intensive care units (PICU) [4], [5], [6]. In this cross-sectional study, we evaluated the impact of CLB including PFS and mechanical valve-type NFC in a pediatric surgery intensive care unit (PSICU).

## Materials and methods

### Study design

This cross-sectional retrospective study was conducted at the Dr. Behçet Uz Children’s Hospital, a tertiary teaching hospital in Izmir, Turkey, between 2021 and 2023. All children aged from 1 day to eighteen years who were followed for more than 48 hours with internal jugular-inserted central venous catheter (CVC) at the pediatric surgical intensive care unit (PSICU) were included. All data was collected from the hospital's infection control committee. 

The study covered two periods: the pre-bundle period from September 1, 2022, to April 30, 2023, when the central line (CL) bundle was not in use, and the post-bundle period from May 1, 2023, to December 31, 2023, after the CL bundle had been in place.

In our PSICU, a non-tunneled catheter with two lumens was used for central venous access. Emergency room catheter insertions were excluded. In the PSICU, insertions into the internal jugular vein were performed using anatomical landmarks to guide insertion during both of the pre-bundle and bundle period. 

### Infection prevention measures in the pre-bundle and bundle period

Prior to insertion, strict hand hygiene, antiseptic skin preparation and aseptic barrier precautions (including mask, gown, protective eyewear, cap, sterile gloves, large or full body drapes, and towels) were taken. 

### Special features during the pre-bundle period

Aqueous povidone-iodine (10% concentration) was used for skin antisepsis, and three-way stopcocks were employed for catheter access. The central line was not routinely flushed.

### Special features during the bundle period

Instead of povidone-iodine, 2% chlorhexidine digluconate in 70% isopropyl alcohol was used. Starting from date of the bundle implementation, healthcare workers participated in weekly education sessions. Prospective and active surveillance as well as compliance monitoring with bundle components (checklist) were carried out throughout the duration of the bundle. Daily inspection of the catheter sites and cap connection, disinfection of the hub with 70% propan-2-ol, use of mechanical valve (BD MaxZero^TM^, Becton Dickinson), and routine flushing of catheter lumens with BD Posiflush^TM^ NaCl 0.9% sterile single-use prefilled syringes (Becton Dickinson) were carried out. The flushing of each lumen of the CL was performed before and after each infusion and blood sampling, and were locked after completion of the final flush.

### Statistical analysis

For each period, the average number of CLABSI episodes per catheter day was calculated. Total catheter days were calculated by summing the total catheter days until the central line was removed. A 95% confidence interval for the incidence rate was used to compute the relative risk ratio and compare the hazards for the two groups. Medcalc 11.6 (Ostend, Belgium) and SPSS version 20 were utilized for statistical analysis (IBM, Armonk, NY, USA). The distributions of numerical variables were examined using analytical and visual methods (histograms and probability graphs). Patient and catheter characteristics were expressed as count (%) or mean value (±standard deviation) for qualitative and quantitative variables. For non-normally distributed quantitative variables, data were expressed as median and interquartile range (IQR). Statistical significance was set at a P-value of 0.05

### Ethics approval and consent to participate

Institutional approval was obtained from the Institutional Review Board of Dr. Behcet Uz Children’s Training and Research Hospital. Informed consent was obtained from the participants (or, in the case of children under 16 years of age, their parents or legal guardians).

## Results

Baseline data: A total of 1092 patients were hospitalized at the PSICU and a total of 142 patients (13.0%) with CVC were included. 81 (57.0%) of the children with CVC were male, and 61 (43.0%) were female. The median patient age was 15 months (range: 1 day to 17 years). Among the patients, 25 were neonates (17.6%) and 94 (66.2%) were younger than 2 years of age. Surgery involved mostly the gastrointestinal system (n=89, 62.7%) and oncological surgery (n=29, 20.4%), followed by thoracic (n=12, 8.5%), the hepato-biliary system (n=11, 7.8%) and burns (1, 28.8%)

### Infection rates

During the pre-bundle period of 509 CVC days, 6 CLABSI were observed with an overall rate of 11.79 CLABSIs per 1,000 CVC days. The microorganisms isolated were *Candida parapsilosis* in 3 cases, *Klebsiella pneumoniae* in 2 cases, and one case each of *Acinetobacter baumannii* and *coagulase-negative Staphylococci*. After bundle implementation, one CLABSI was diagnosed in 621 CVC days with an overall rate of 1.61 CLABSIs per 1,000 CVC days. The CLABSI rate was thus significantly lower in the bundle period (p= 0.03) (Table 1 (Tab. 1)). 

## Discussion

In this study, the effectiveness of a CVC bundle was evaluated by comparing data from time periods before and after bundle implementation in the PSICU. The CLABSI rate decreased dramatically and statistically significantly from 11.79 per to 1.61 per 1,000 CVC days, with a ten-fold decrease after implementation of a CVC bundle that included NFC and PFS.

Despite the often-reported fact that surgical interventions are associated with risk of developing healthcare-associated infections including CLABSIs, studies conducted on PSICUs are few [7], [8], [9], [10]. In our center, the rate of CVC use in total hospitalized patients at the PSICU was 13.0%, which was relatively low compared to the reports from non-surgical PICUs, which had a 73.0% usage rate [11]. Reports including CVC-related infectious complications in pediatric surgical patients are relatively limited. In one study from India, CVC-related bloodstream infections were reported to be 17.4/1,000 catheter days in pediatric surgery patients [12]. In addition, Rao et al. [13] found a CVC-associated infection rate of 15.4% (11/1,000 line days) in a pediatric surgical unit. 

The efficacy of CLB programs for prevention of CLABSIs have been reported for different intensive care units [4], [5], [6], [14], [15], [16], [17]. Moreover, in a recent study from our center focusing on patients in PICU, a CLB was found to be effective in decreasing the CLABSI rate, daily hospital costs, and antimicrobial drug expenditures in children [6]. Although previous studies were mostly focused on pediatric intensive care units, studies focusing on pediatric surgery ICUS are rare, if pediatric cardiovascular surgery ICUs are excluded [18], [19].

Needle-free connectors, which are also referred to as closed systems, were introduced into clinical practice with the purpose of preventing the use of needles on intravascular catheters and aimed to replace the 3-way 3WSCs (also defined as open systems) in clinical practice [20]. Needle-free connectors include split-septum connectors and luer-activated mechanical valves. One study from an adult surgery ICU reported a significant decrease at the incidence of CVC- related bloodstream infections by using BD MaxZero^TM^ [21]. At our center, the positive impact of CLB – in which one of the important bundle elements is split-septum type NFCs – has been reported in different clinical settings, but this study is the first study in Turkeyto include BD MaxZero^TM^ usage in children. In another study, Wallace et al. [22] reported that CLABSI rates decreased to 0.91 from 4.8/1,000 catheter days with the implementation of MaxPlus^TM^, which has the same structural properties. The previous reports on the impact of NFCs including mechanical valves support our findings [22], [23], [24], [25], [26], [27].

Flushing and locking are crucial elements of CLB, because flushing of the catheter prevents intraluminal occlusion and formation of biofilm, while also preventing blood reflux [28], [29], [30]. As reported earlier, the implementation of flushing, including PFS, in addition to NFCs reduces CLABSI [4], [5], [6], [31], [32]. The risk of contamination with different pathogens with manually prepared saline for flushing was reported to be 6 to 16% [33], [34], [35]. Ceylan et al. [36] reported that there were significantly more failed attempts in the manually flushed group, which may increase the risk of infection, compared to the group using single-use sterile pre-filled saline syringes.

## Limitations

This study has limitations due to its design. First, data were collected retrospectively from medical files and hospital data systems. Moreover, the authors did not have a computerized surveillance system for bundle compliance at their disposal. In our hospital, infection control nurses and clinicians visited the PSICU daily and recorded the catheter days as well as development of the catheter infections, and entered this data into our calculating system. In addition, we could not measure the effect of each bundle element individually because we implemented all CLB elements simultaneously.

## Conclusion

A CLB including PFS and NFCs significantly reduced CLABSI rates in our PSICU. Moreover, in hospitals with pediatric surgical ICUs y as well as surgical ICUs for other patient groups, a bundle approach to prevention infections should be implemented. 

## Notes

### Authors’ ORCIDs

- Devrim I: https://orcid.org/0000-0002-6053-8027
- Ergun D: https://orcid.org/0000-0002-6069-1478
- Demet Payza A: https://orcid.org/0009-0002-9121-775X
- Ozbakir H: https://orcid.org/0000-0003-0721-3511
- Boztas Demir AE: https://orcid.org/0000-0002-5663-9394
- Kaçar P: https://orcid.org/0000-0002-2669-419X
- Can M: https://orcid.org/0000-0002-4263-0911
- Bayram N: https://orcid.org/0000-0003-1802-2518
- Sencan A: https://orcid.org/0000-0003-4499-7984

### Funding

None. 

### Competing interests

The authors declare that they have no competing interests regarding this study.

## Figures and Tables

**Table 1:**
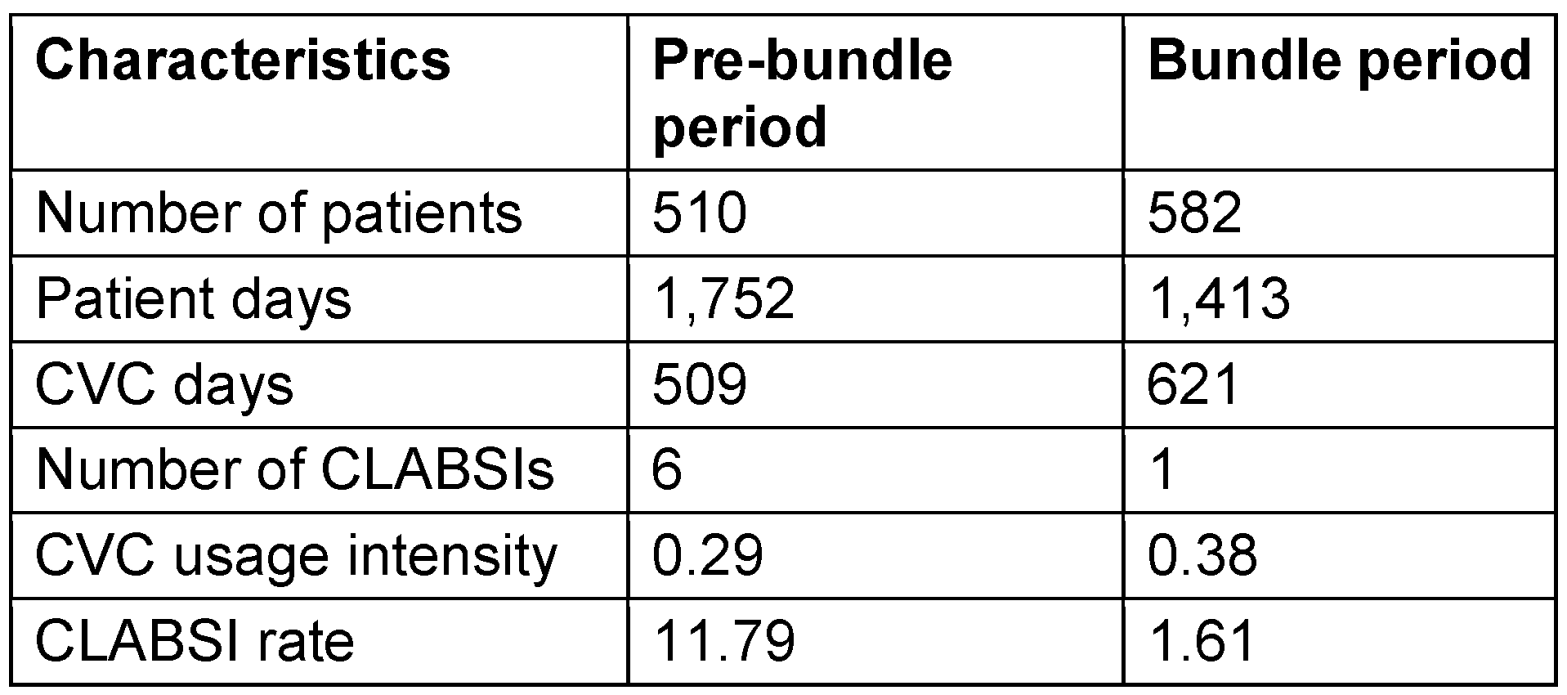
CVC-associated bloodstream infection rates during the study period
